# The Potential of Probiotics as Ingestible Adjuvants and Immune Modulators for Antiviral Immunity and Management of SARS-CoV-2 Infection and COVID-19

**DOI:** 10.3390/pathogens12070928

**Published:** 2023-07-11

**Authors:** Sophie Tomkinson, Cloe Triscott, Emily Schenk, Andrew Foey

**Affiliations:** 1 School of Biomedical Sciences, Faculty of Health, University of Plymouth, Drake Circus, Plymouth PL4 8AA, UK; stomkinson1701@outlook.com (S.T.); cloetriscott@sgmail.com (C.T.); emily.schenk@students.plymouth.ac.uk (E.S.); 2 Peninsula Medical School, Faculty of Health, University of Plymouth, Drake Circus, Plymouth PL4 8AA, UK

**Keywords:** probiotics, antiviral immunity, SARS-CoV-2, COVID-19, immune evasion

## Abstract

Probiotic bacteria are able to modulate general antiviral responsiveness, including barrier functionality and innate and adaptive immune responses. The COVID-19 pandemic, resulting from SARS-CoV-2 infection, has created a need to control and treat this viral infection and its ensuing immunopathology with a variety of approaches; one such approach may involve the administration of probiotic bacteria. As with most viral infections, its pathological responses are not fully driven by the virus, but are significantly contributed to by the host’s immune response to viral infection. The potential adoption of probiotics in the treatment of COVID-19 will have to appreciate the fine line between inducing antiviral immunity without over-provoking immune inflammatory responses resulting in host-derived immunopathological tissue damage. Additionally, the effect exerted on the immune system by SARS-CoV-2 evasion strategies will also have to be considered when developing a robust response to this virus. This review will introduce the immunopathology of COVID-19 and the immunomodulatory effects of probiotic strains, and through their effects on a range of respiratory pathogens (IAV, SARS-CoV, RSV), as well as SARS-CoV-2, will culminate in a focus on how these bacteria can potentially manipulate both infectivity and immune responsiveness via barrier functionality and both innate and adaptive immunity. In conclusion, the harnessing of induction and augmentation of antiviral immunity via probiotics may not only act as an ingestible adjuvant, boosting immune responsiveness to SARS-CoV-2 infection at the level of barrier integrity and innate and adaptive immunity, but also act prophylactically to prevent infection and enhance protection afforded by current vaccine regimens.

## 1. Introduction

Probiotics and their potential therapeutic use as an immune enhancer or immune regulator is still relatively novel and unexplored. Past investigations have demonstrated that bacterial probiotics support a healthy bacterial microbiome, thereby maintaining gut mucosal barrier integrity as well as reducing the risk of infection. This natural and easily attainable immuno-modulatory bacterial source has been found to increase the body’s antiviral immunity as well as modulate pathogen-stimulated inflammation [1]. The direct mechanisms by which these probiotic species and strains affect antiviral immune responses, however, are yet to be fully explored.

Respiratory infections, such as pneumonia and influenza, contribute significantly to the annual worldwide death toll. Further to this, respiratory associated pandemics have a detrimental effect upon the economy, due to the rise in hospitalizations and cost of patient care. In the past two decades, there have been six significant infectious global outbreaks, with four of these resulting in mucosal respiratory tract infections, namely: severe acute respiratory syndrome coronavirus, or SARS-CoV (2002–2004); H1N1 influenza (2009–2010); the Middle East Respiratory Syndrome coronavirus, or MERS-CoV (2012–2020); and SARS-CoV-2 (2019-present) [2]. These regular eruptions of viral pandemics are due to various factors such as the daily movement of people, climate change, limited selection of available antiviral agents, increased number of individuals suffering from co-morbidities which elevate the number of immunocompromised subjects, and the genetic advancement of viral immuno-evasive mechanisms.

To date, the viral family *Coronavirdae* has been found to have seven human strains: NL63, 229E, HKU1, OC43, SARS-CoV, MERS, and the 2019 novel coronavirus [3], of which SARS-CoV and SARS-CoV-2 belong to the genus *Betacoronavirus*. SARS-CoV-2, which causes COVID-19 pathology, is a positive-sense enveloped RNA virus, with distinctive crown-like ‘spikes’ projecting from its capsid surface, and is the most recent highly infectious pandemic to date [4]. SARS-CoV-2 infection symptoms vary significantly from mild symptoms to death. Most commonly, a dry cough, fever, loss of taste and/or smell and shortness of breath are experienced. Many individuals also felt extremely lethargic, both during and post-infection, and had a continuous feeling of throat and muscle pain [5]. Long-term sequelae of SARS-CoV-2 infection can include severe organ damage, which occasionally leads to organ failure, as well as secondary infections, such as pneumonia caused by opportunistic bacteria [6,7]. The long-lasting and highly detrimental effects caused by SARS-CoV-2 infection are predominantly due to the cytokine storm (or cytokine release syndrome, CRS) thereby elicited, where there is a significant induction and secretion of pro-inflammatory cytokines, such as IL-6 and TNF-α. IL-6, in particular, has been suggested as a potential therapeutic target for acute respiratory distress syndrome (ARDS) in SARS-CoV-2-infected COVID-19 patients [8]. In addition, Tocilizumab, which antagonises IL-6 function by targeting IL-6R, has been shown to beneficially affect survival and clinical outcomes in the treatment of COVID-19 patients with severe pneumonia [9,10].

It is now well established that the microbiome of the gastrointestinal tract (GIT), and its supplementation with exogenous probiotic strains, has a direct effect upon not only mucosal barrier integrity, but also the immunological status of a patient. Depending on the probiotic bacterial strain consumed, the host’s immune system can be suppressed or regulated, deviated from one type of immune response to another, or augmented by strengthening innate responses and/or adaptive humoral responses against exogenous pathogens or adaptive cell-mediated immunity to intracellular-resident pathogens and tumours [11]. Thus, probiotic supplementation may be harnessed to optimise antiviral immune responses, offering a realistic protective regimen against SARS-CoV-2 infection and pathogenesis [12]. This review will focus on the potential beneficial effects of probiotic bacterial supplementation on antiviral immunity, with a particular emphasis on respiratory viral pathogens and the future adoption of probiotics in the prophylactic and treatment control of SARS-CoV-2 viral infection associated with COVID-19.

## 2. Gut Mucosal Immune Function and Influence of Commensal Microbiome and Probiotics

### 2.1. The Gut Microbiome

The human microbiome consists of approximately 10^14^ microbes, including bacteria, archaea, eukarya, and viruses, with an estimated 100 trillion of these inhabiting the gut [13]. This vast number of commensal microbiome includes over 1000 species-level phylotypes, including over 400 identified species [14], with the majority being firmicutes (e.g., *Clostridium* and *Bacillus*) and bacteroidetes (e.g., *Bacteroides*), as well as notable numbers of Proteobacteria (e.g., *Escherichia*) and Actinobacteria (e.g., *Bifidobacterium)* [15]. Aside from preventing infection and pathogenic colonisation of the gut by outcompeting invaders and affecting their ability to infect the host via modification of virulence factors [16], the presence of these bacteria influences immune function, and it has been suggested that the presence of these organisms is crucial for an effective immune response of the GIT and peripheral tissues.

### 2.2. Structure–Functionality of the Gut Mucosa

**Figure 1:**
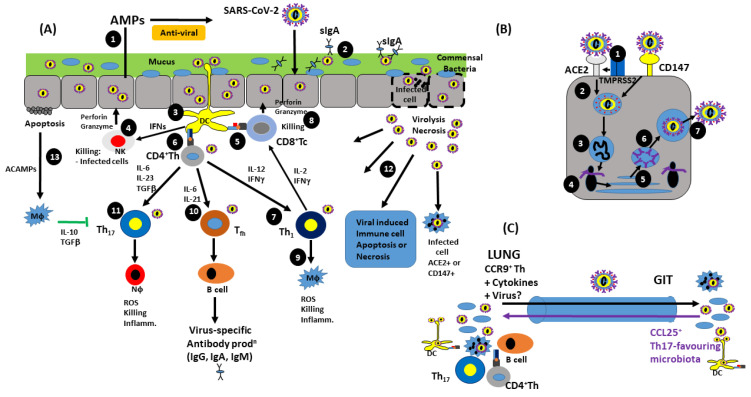
**Host antiviral immune response to SARS-CoV-2 infection: recognition, immunity and the gut–lung axis.** (**A**) IR to SARS-CoV-2 infection: infected mucosal epithelial cells elicit several responses which include (1) secretion of AMPs, (2) Ab-mediated neutralisation, (3) DC-Ag processing and presentation, (4) NK cell activation and killing of virus-infected cells, (5) APC-MHC I-Ag presentation and activation of cytotoxic T cells, (6) MHC II-Ag activation of naïve Th cells, (7) cytokine-driven Th_1_ differentiation which activate (8) Tc killing of virus-infected cells and (9) DTH activation of Mϕ inflammation and killing responses. (10) Cytokine differentiation and activation of Tfh, resulting in B cell Ab production, (11) cytokine differentiation and activation of Th_17_ and Nϕ activation–inflammation. (12) Virolysis/cell necrosis resulting in immune activation and (13) virus-induced apoptosis and ACAMP-induced anti-inflammatory cytokine production to limit antiviral responsiveness. (**B**) Reception, infection, and viral replication. SARS-CoV-2 spike protein binds to surface viral receptors ACE2 (grey) and CD147 (yellow). Upon S protein binding to ACE2, (1) TMPRSS2 protease (membrane-bound blue boxes) cleaves S protein, allowing S2 facilitation of virus envelope fusion with the cell membrane; (2) virus entry in endosomal compartment and (3) release of +sense strand vRNA which is (4) transcribed by ribosomes and (5) post-ER and Golgi processing of viral proteins, leading to (6) virion construction and (7) release of virus from infected cell by exocytosis. (**C**) Gut–lung axis of viral, microbiome, and immune cell/molecule transport. Mucosal infection of lung tissue can result in viral translocation to the GIT, which primes dysbiosis of the microbiome and influences IR and Th_17_ bias, resulting in CCL25 and CCR9 transport from GIT to lung. CCL25 chemoattracts CCR9^+^ cells, which also include Mϕs, DCs, IELs and IgA^+^ B cells.

The GIT is comprised of an intestinal mucosal surface; a single epithelial cell layer that contacts and interacts with intestinal lumen contents and that overlies the lamina propria [17]. The epithelial cell layer is mainly formed of cells derived from intestinal epithelial stem cells, differentiated to become enterocytes (the most abundant cell type), goblet cells (mucin production centres), paneth cells (responsible for secretion of antimicrobial defensins), enteroendocrine cells (involved in hormone release and digestion regulation), and microfold (M) cells (which are found in the folds of the microvilli and which deliver antigens to immune cells) [17]. As well as these epithelial cells, immune cells such as intraepithelial lymphocytes (αβ and γδ TCR^+^ T cells) and dendritic cells (DCs) are also found in the epithelium [17,18,19]. As such, the lamina propria is the main site of immune induction in the gut mucosa. Aside from structural cells such as fibroblasts, smooth muscle cells, and vascular endothelial cells [20], the tissue also contains several types of immune cell, including dendritic cells, T cells, B cells and macrophages (Mϕs) [19,21] (refer to Figure 1a). The major difference between the small and large intestine is the presence of specialised immune structures called Peyer’s patches in the small intestine [22]. These are peripheral lymphoid tissues comprised of segregated populations of T cells and B cells as well as DCs [23]. M cells, located in the follicle-associated epithelium (FAE) dome region, overlie the Peyer’s patches (PP) and introduce naïve lymphocytes to antigens to induce differentiation, resulting in antigen-specific immune competent cells [23]. Other lymphoid tissues found in the lamina propria of the gut are isolated lymphoid follicles, cryptopatches, and colonic patches (found only on the large intestine) [24]. The other difference between the two intestinal types is the mucin-producing goblet cell composition of the mucosal layer. In the small intestine, there is a more widely spaced distribution of goblet cells, leading to a broken and less-developed mucus layer, to facilitate nutrient absorption. In the large intestine, a higher density of goblet cells leads to a thicker continuous mucous layer that is more adept at compartmentalising pathogens and retaining molecules such as IgA and antimicrobial peptides (AMPs), which are involved in defence against pathogenic microbes [25].

The complex array of cells involved in enteric immune function allows the host to effectively fight off viral pathogens. Interestingly, it is not just enteric viral pathogens that are capable of stimulating the gut immune response; viruses causing infection in other sites, such as the lungs and respiratory tract, can elicit an immune response from the gut mucosa [26]. The interaction between the gut and lungs is referred to as the ‘gut–lung axis’ and has been found to play a role in shaping the respiratory immune response to viral pathogens [27] (refer to Figure 1c). This interaction is thought to be largely influenced by the gut microbiome, where a study by Schuijt et al. (2015) [28] demonstrated that antibiotic-treated mice (i.e., those showing depletion/alteration of the gut microbiota) were more susceptible to bacterial dissemination, inflammation, organ damage, and death when infected with the respiratory bacterial pathogen *Streptococcus pneumoniae*. This study also found that microbiome-depleted mice displayed altered alveolar macrophage (AM) function and diminution of phagocytic ability. Further to the gut–lung axis influencing respiratory viral infection responses via microbiome-stimulated systemic immune responses, it is also possible for respiratory viruses to directly infect enteric cells possessing the appropriate viral receptors. Indeed, infection by H5N1 avian influenza A virus is mediated by SA-α2-6-gal receptors, which can be found on gut enterocytes [29], through which H5N1 was capable of infecting and replicating in ex vivo human colonic tissue. In addition, H5N1 influenza A viral antigens were also found in gut biopsies, reinforcing enteric involvement of this respiratory infection and the ‘gut–lung axis’. Understanding how respiratory viruses infect the gut is particularly pertinent in consideration of the current COVID-19 pandemic, with up to 50% of patients possessing detectable levels of SARS-CoV-2 viral RNA in their faeces, even when rhino-pharyngeal swabs produce negative test results [30].

Studies in gnotobiotic (germ-free) mice or those treated with antibiotics (i.e., their gut flora were diminished) show poor immunological function compared to those with a healthy microbiome. Not only does a lack of enteric bacteria lead to increased susceptibility to infections of the gut [31], but has also been found to impair the development of lymphoid tissues such as Peyer’s patches and reduce T cell count in the mucosal immune system [32]. As well as influencing the response to intestinal infection, a healthy gut microbiome confers improved immune response to infections at other sites. Mice treated with broad-spectrum antibiotics for 2 weeks (ABX) were shown to take significantly longer to clear infection of blood pathogen Lymphocytic choriomeningitis virus (LCMV) compared to control subjects. As well as this, ABX mice were found to have significantly reduced LCMV-specific CD8^+^ T cell and IgG antibody titres and less efficient production of cytokines involved in the viral immune response, such as IFN-γ, TNF-α, IL-2, and MIP-1α [31].

The microbiome also affects the systemic immune system in multiple ways. Firstly, the intestinal microbiome mediates the expansion and differentiation of extra-intestinal T cells as demonstrated in mice, where polysaccharide A (PSA) produced by lower-GIT coloniser *Bacteroides fragilis* lead to enhanced CD4^+^ T cell activation and correction of T cell deficiency and Th_1_/Th_2_ imbalances seen in germ-free counterparts [33]. Furthermore, interactions between commensal gut bacterial microbial associated molecular patterns (MAMPs), such as lipopolysaccharide (LPS) and peptidoglycan, and toll-like receptors (TLRs) stimulated NF-κB activation and downstream expression of several pro-inflammatory genes. In addition to anti-pathogen responses, this interaction is crucial to maintaining an adequate TLR response, allowing for activation of CD4^+^ and CD8^+^ T cells, antibody production, activation of the inflammasome, and DC migration [34].

### 2.3. Probiotic-Derived Metabolites Modulate Host Immunity

This influence of the gut mucosal immune system occurs through the direct effect of endogenous commensal or exogenously sourced probiotic microbes on immune cell function or indirectly as a consequence of synthesis of immunomodulatory molecules [18]. Short-chain fatty acids (SCFAs) such as acetate, propionate, and butyrate, metabolised by both gut commensal and probiotic bacteria during anaerobic breakdown of fibre, e.g., by commensal bacteria from *Clostridium butyricum* species and probiotic bacteria (*Lactobacillus* and *Bifidobacteria*), are key regulators of the intestinal immune response. SCFAs exert a wide range of modulatory effects on immunological cells and signalling pathways by binding to G-protein-coupled receptors (GPRs), primarily GPR43, GPR41, and GPR109a [35]. Butyrate reception, via GPR109A, supresses NF-κB, leading to M2 Mϕ (anti-inflammatory/regulatory phenotype) polarisation, with Mϕs showing increased activation of the Histone 3 Lysine 9 (H3K9)/signal transducer and activator of transcription 6 (STAT6) signalling pathway [36,37]. Additionally, M2 polarisation is further reinforced by Butyrate-GPCR induction of prostaglandin E_2_ and anti-inflammatory IL-10 [38,39]. Conversely, the interaction between GPR43 and acetate leads to increased production of IL-18, a cytokine key in intestinal repair and cell-mediated immunity to viral infection [40]. SCFAs can also directly influence gene expression by inhibiting histone deacetylase (HDAC) activity, independent of GPCRs [41]. In colonic lamina propria Mϕs, butyrate is associated with increased H3K9 acetylation, leading to decreased pol II and pol II S5P (RNA polymerases) recruitment to the promoter regions of pro-inflammatory cytokines IL-6 and IL-12, and Nitric Oxide Synthase 2 (NOS2), hence exerting an anti-inflammatory effect [41]. SCFAs also exert pro-inflammatory effects on immune cells of the gut. Butyrate-mediated HDAC inhibition was found to upregulate CD8^+^ cytotoxic T cell (Tc) production of IFNγ and granzyme B [42], thereby augmenting cell-mediated immunity against intracellular pathogens. Thus, the balance of commensal microbes with potential pathogenic microbes and the influence of exogenous sources of probiotics is vital to the ability of both gastrointestinal and respiratory/lung mucosal immune systems to mount and control appropriate immune responses.

## 3. Immunomodulatory and Antiviral Capability of Probiotics

Probiotics are live, non-pathogenic species of microbes that confer a realistic health benefit on the host, which in combination with prebiotics (indigestible dietary fibre/carbohydrates), confer health benefits, including immunomodulation, directly via microbe-host cell interactions or indirectly via products resulting from anaerobic fermentation [43]. Probiotics are generally administered to the host via ingestion of dairy products such as yogurts containing common probiotic organisms including *Lactobacillus*, *Bifidobacterium*, *Enterococcus*, *Leuconostoc* and *Pediococcus* [44]. In general, they have been demonstrated to influence health and response to infection when their impact on the gut microbiome allows for a more effective immune response to pathogens [45,46].

Probiotics have been demonstrated to exert a wide variety of effects, which would appear to be both immune activatory and regulatory, on the immune system dependent on probiotic species and the strain being ingested (reviewed by Hardy et al., 2013 [11]). Starting with the barrier epithelial cells of the GIT, *B. subtilis* OKB105 and *B. adolescentis* LMG10502 competitively inhibit viral adherence, and hence infectivity, of gastroenteritis coronavirus and norovirus [47,48]. This mucosal barrier influence extends to the induction of AMPs, whereby *L. casei* DN-114001 enhanced defensin expression in response to RTI, rhinopharyngitis, and influenza [49]. One of the most consistent and powerful innate responses against viral infection is to prevent its replication and hence perpetuation of infection. This is achieved via the induction of Type I IFNs; probiotics (*L. plantarum* L137, *L. rhamnosus* GG, *L. rhamnosus* CRL-1505/1506, *L. casei* Shirota, *L. brevis* KB-290, *L. pentosum* b240 and *L. lactis* JCM5805) have been demonstrated to effectively induce and enhance the production of these antiviral cytokines in response to influenza (H1N1) and RSV infection [50,51,52,53,54,55,56,57,58,59,60,61]. In addition, natural killer (NK) cells both produce IFNs and are responsive to these antiviral cytokines. NK cells detect virally infected cells via their capacity to recognize viral-induced changes in expression levels of MHC Class I molecules or their structural modification, resulting in NK-directed cytotoxic responses targeted at both the virus directly and the host cell that the virus replicates inside. Probiotic microbes have also been shown to increase both NK numbers and killing functionality, with LcS and *L. delbrueckii* spp. bulgaricus OLL1073R-1 potently enhancing clearance of CMV and EBV [62,63,64,65,66]. With regard to modulation of inflammatory responses, probiotics containing *L.plantarum* and *Lacticaseibacillus casei* Shirota may exert both pro-inflammatory effects through stimulation of IL-6 production and anti-inflammatory effects via suppression of IL-12 in Peyer’s patch cells and mucosal Mϕs, whereas probiotics containing *B. bifidum* may induce an anti-inflammatory effect by suppressing the pro-inflammatory cytokine TNF-α and inducing the anti-inflammatory cytokine IL-10. Indeed, *L. plantarum* strains (L137, CNRZ1997, NCIMB8826) have all been demonstrated to induce pro-inflammatory effects protective against H1N1 and RSV infection [67,68,69,70], some of which are TLR-dependent [70]. As a consequence of these strain-dependent effects modulating both pro- and anti-inflammatory cytokines, these probiotic cytokine responses not only loop back to induce NK cell activity but can modulate antigen-specific antiviral adaptive immune responses by enhancing Th_1_ and Th_2_ cell numbers and functionality as well as Treg differentiation for immunomodulatory/suppressive effects [71].

Adaptive antiviral immune responses are driven by both cell-mediated immunity (CMI) and humoral responses. CMI responses result in the activation of cytotoxic T cells (Tc) and/or delayed-type hypersensitivity (DTH) responses, whereby IL-12-differentiated Th_1_ cells activate pro-inflammatory Mϕ responses via Th_1_-produced IFNγ. Humoral responses result in B cell production of antiviral antibodies which, dependent on isotype, drive viral neutralisation of infectivity (IgA) or lysis of virus-infected host cells via ADCC (IgG). The vast majority of reports of probiotic enhancement of CMI responses have utilized both human patient data and experimental infection-challenge animal model data. In general, *L. plantarum* L137 reduces URTI via the upregulation of IL-12 expression [50,72,73], whereas *L. plantarum* L136, *L. plantarum* YU, *L. fermentum* LF1, *L. fermentum* CJL-112, and *L. gasseri* TMCO356 all reduce H1N1 infection via induction of IL-12 or Tc/DTH responses [51,67,68,70,74,75]. Probiotic bacteria have also been shown to enhance humoral antibody-mediated immunity by increasing the expression and secretion of sIgA, which neutralizes infectivity and is associated with reduced H1N1 influenza viral loads in response to *L. fermentum* LF1, *L. fermentum* CJL-112, *L. gasseri* TMCO356, *L. brevis* KB-290, *L. paracaseii* ssp. paracasei, and *L. casei* 431R [51,54,74,75,76].

In addition, metabolites such as SCFAs and other probiotic-derived products may also modulate immune mechanisms with an established anti-inflammatory effect [38]—useful when considering viral infection that induce harmful, tissue-destructive pro-inflammatory host responses. Indeed, probiotics have a beneficial role in the assistance of SCFA transport. Borthakur et al. (2010) [77] found that *L. plantarum* suppressed TNF-α inhibition of sodium-coupled monocarboxylate transporter (SMCT1), a molecule associated with SCFA cross-membrane transport, thereby allowing SCFAs to have a stronger influence and modulate the immune response, potentially looping back to suppress TNF-α production and induce production of the anti-inflammatory IL-10.

Probiotic effects are not limited to the gut and have been shown to be involved in protection against respiratory pathogens. In addition, probiotic administration of *B. breve*, *L. pentosus,* and *L. brevis* influence the immune response to the respiratory virus influenza, leading to improved IgG and IgA production, as well as reduction in viral titres and issues related to influenza such as weight loss and alterations of physical condition [78]. Other effects on the immune system seen from probiotic introduction include increased polymorphonuclear cell recruitment, phagocytosis, and TNF-α and IgA production following administration of different *L. paracasei* strains [79]. Probiotics including *Lactobacillus* and *Bifidobacterium* species have also been found to increase the cytotoxic effect of NK cells and influence the production of many other key pro-inflammatory cytokines, including IL-1β, IL-4, IL-5, IL-6, IL-8, and IL-13 [43]. Due to this broad effect on the immune system, it is theorised that probiotics may be beneficial in the response to COVID-19 [44], both as a prophylaxis to boost immune functionality and prevent infection and as a partial therapeutic in the treatment of SARS-CoV-2-infection.

## 4. COVID-19: Infection by SARS-CoV-2

Approximately 80% of patients who have recently contracted COVID-19 suffer only from mild symptoms such as fever, cough, and headaches. Infection can, however, cause a large variety of symptoms in individuals, from being asymptomatic to having a chronic infection which can lead to organ damage and secondary opportunistic infections [80]. Initial infection by SARS-CoV-2 is through mucosal surfaces, such as the respiratory tract and the GIT [81]. Infection of the nasopharynx and trachea occurs via inhalation, whilst infection of the stomach arises via ingestion. The discovery that SARS-CoV-2 has potential as an enteric pathogen comes as little surprise following research into previous coronavirus outbreaks, namely SARS-CoV, with which SARS-CoV-2 shares around 80% of its viral genome [82,83], and MERS-CoV, which also showed evidence of enteric infection, with around 30% of MERS-CoV patients and 10.6% of SARS-CoV patients presenting with diarrhoea [84]. There have been several cases reported where gastrointestinal symptoms appeared before respiratory symptoms, with patients developing diarrhoea. For example, the first-ever individual to develop symptoms in the United States was nauseous and had symptoms such as vomiting for approximately two days before being admitted to hospital and upon admission developed diarrhoea. Building on the known prevalence of gastrointestinal symptoms in SARS-CoV-2 infected patients and the potential of a gut–lung axis of infection and immune defence, further investigation is warranted as it may provide an alternative delivery of treatment targeting the respiratory tract [81].

SARS-CoV-2 is a single-stranded positive sense RNA genome virus of 30 Kb, which is associated with a phosphorylated nucleocapsid protein contained in an enveloped phospholipid bilayer organised in a spherical shape 80–120 nm in diameter. The viral genome encodes 28 proteins, of which 16 are non-structural proteins (Nsp 1–16), 4 structural (S, spike; M, membrane; E, envelope; N, nucleocapsid), and 8 accessory proteins. In the case of COVID-19, viral infection occurs via binding of glycosylated viral spike (S) proteins to angiotensin converting enzyme 2 (ACE2), regulated via cleavage of S protein S1 and S2 domains by transmembrane protease serine protease 2 (TMPRSS2) [85]. It must be noted, however, that CD147 has also been reported to bind to the spike protein and facilitate viral entry/infection [86]. Currently, it is not clear as to whether this receptor is an alternative receptor or acts as a co-receptor to ACE2. Although the primary infection site is the respiratory tract, ACE2 receptors are also found on gut enterocytes in the small intestine [87] and the colon [88], with research suggesting higher protein expression levels in the gut [80,89]. As with SARS-CoV-2, SARS-CoV infects host cells by binding to the ACE2 receptor [90], so hypotheses about how SARS-CoV-2 operates in the human body can be formulated based on how SARS-CoV behaves. As there is a clear intestinal involvement with both viral infections due to ACE2 expressed on enterocytes, there is potential for the gut microbiota and thus probiotics to affect disease progression, by either inducing a protective immune response or modulating ACE2 expression and hence infectivity. This is indicative of probiotics being adopted for prophylaxis, treatment, and optimisation of vaccine use.

SARS-CoV-2 surface spikes can be classified into two main types, S1 and S2, respectively. The S1 region, which consists of one N-terminal domain and three C-terminal domains (CTD1, CTD2, and CTD3) is required for host cell receptor attachment by CTD1 specifically to ACE2 receptors. The S1 protein, in particular its receptor-binding domain (RBD), is heavily glycosylated and is the most variable structure in coronaviruses. The S2 protein, on the other hand, causes membrane fusion between viral envelope and host cell membrane to allow viral entry to the cell cytosol, where viral replication soon follows [91,92]. Construction of new virions are produced by replicating genomic information, followed by budding or secretion from the cell as a newly constructed SARS-CoV-2 virus [93] (refer to Figure 1b).

## 5. Antiviral Immunity and SARS-CoV-2 Immunopathology

### 5.1. Innate Immunity

Initially, infection is dealt with via the innate immune response, which becomes stimulated after only a couple of hours [94]. In general, innate responses to viral infection involve several phases which will be dealt with in the following order and include: (1) Mucosal barrier responses (e.g., mucus production), expression of anti-microbial peptides (AMPs) and junctional integrity between epithelial cells; (2) detection of PAMPs and DAMPs: TLRs (TLR-3, -7, -8, -9), NLRs (inflammasomes), and RLRs (RIG-1, Mda-5, Mavs etc.); (3) Cellular recruitment and activation (NKs, Mϕs, Neutrophils, DCs); (4) secretion of Type I and II IFNs, ROS, RNS, hydrolytic enzymes, and complements; (5) production, secretion, and signalling of innate cytokines—pro- vs. anti-inflammatory cytokines (refer to Figure 1).

Although the clear relationship between mucosal AMPs and responsiveness to SARS-CoV-2 infection has not been adequately established, several observations suggest that AMPs play a role in innate responsiveness to this virus. With regard to immunity to IAV infection, the production of Cathelicidin (LL37) has been observed to inhibit IAV infection via disruption of the viral envelope [95]. In addition, IAV (H1N1, H3N2 and H5N1) induces Nϕ bactericidal/permeability-increasing protein (BSI), which is capable of inhibition of IAV infection of lung epithelial cells and destruction of the viral envelope [96]. Upon investigation of the antiviral effect of mouse β-defensin-4, a short peptide, P9, was found to exhibit antiviral effects against the respiratory virus, IAV, MERS-CoV, and SARS-CoV through its ability to bind viral glycoprotein P9 to prevent endosomal acidification, thus blocking membrane fusion and viral RNA release [97]. Finally, the rhesus theta-defensin-1 (RTD-1) AMP, used in a murine model of SARS-CoV pulmonary disease, acted prophylactically to prevent both death and altered lung tissue cytokine responses, effectively exerting an immunomodulatory effect [98]. Collectively, when considering involvement of mucosal cells such as paneth cells and Nϕs, the use of Defensin-5 has been suggested as a competitive inhibitor of SARS-CoV-2 binding to ACE2, hence preventing viral infection [99].

Inflammation is one of the key responses when dealing with a SARS-CoV-2 infection. Phagocytes, such as Mϕs and Nϕs, are recruited to the lungs and detect the virus using pattern recognition receptors (PRR), such as the toll-like-receptors (TLR) TLR7/8 and TLR4, which detect viral single-stranded RNA and the spike protein, respectively [100,101]. TLR7 binds the SARS-CoV-2 E protein whereas TLR8 binds Nsp8 RNA fragments. Activation of these PRRs induces an extensive cytokine profile, wherein TLR8 induces IL-1, IL-6, IL-8, MIP-1α, MIP-1β, IL-12, and TNF, whereas TLR7 induces higher levels of IFNα, CXCL10, and I-TAC [102,103,104]. It is important to note that dysbiosis of the gut microbiota resulting from IAV infection influences TLR7 signalling, whereby mRNA levels of TLR7, Myd88, IRAK-4, TRAF6, and NFκB are reduced [105]. Such dysbiotic reductions in these signalling molecules will severely hinder viral recognition and the downstream antiviral innate effector responses. TLR activation initiates a signalling cascade which results in the nuclear translocation of the transcription factor NFκB to induce expression of the pro-inflammatory cytokines IL-1β, TNF-α, and IL-6 [101], whereas Mϕ NLRP3 inflammasome activation via viral ORF3a induces the caspase-dependent maturation and secretion of the pro-inflammatory cytokine IL-1β as well as the NK cell activator, IL-18 [106]. This raises the possibility that the inflammatory response is both beneficial and detrimental to host and pathogen. These cytokines augment neutrophil and monocyte recruitment. Indeed, neutrophilia is one of the hallmarks of COVID-19 immunopathology, which, alongside an over-exuberant pro-inflammatory cytokine storm (CRS), was found in 38% of patients with CXCR1, siglec5, CD177 and the antimicrobial peptide DEFA1 upregulation in severe COVID-19 patients [89,107]. In milder COVID-19 cases, the inflammatory response is beneficial and aids viral clearance, whereas in severe cases, persistent chronic inflammation can contribute to significant tissue damage and organ failure [108], responses similar to those observed in sepsis.

Another product of PRR-mediated viral recognition in alveolar macrophages (AMs) is the induction of type I and type III interferons (IFN) [109]. Whilst both IFNs induce a strong antiviral state in neighbouring cells, the effect of type I IFN is much more global compared to type III IFN, which is limited to mucosal surfaces of the respiratory and gastrointestinal tract [110]. These antiviral effects follow the expression of IFN-stimulated gene products which include CXCL10 (IP-10: chemotactic for Th_1_, Th_2_, NK and B cells) and RNAse L (vRNA destruction and MDA5-dependent induction of IFNβ expression leading to host cell autophagy and apoptosis, [111]). However, SARS-CoV-2 ORF3b can antagonize IFN production, effectively delaying the host’s innate antiviral response and allowing for increased viral replication [100] (refer to evasion strategies in Table 1). NK cells are activated by both TLR7 and IFNα, the knockout of which suppresses IFNγ and granzyme production [112,113]; these cells play a role in antiviral immunity during SARS-CoV-2 infection, Where The Spike Protein Increases NK chemotaxis [114]; their numbers however, seem to be outweighed by the presence of neutrophils recruited to the site of infection. Since NKs function similarly to Tc in eliminating virally infected cells, it is possible that NK count, and indeed its relative abundance ratio to neutrophils, might serve as another diagnostic determinant for mild and severe pathology, with greater neutrophil abundance being indicative of a poorer prognostic outcome.

Another molecular mechanism important in determining antiviral or host tissue-destructive responses is the respiratory burst, resulting in the production and secretion of ROS/RNS. Indeed, rapid ROS production is associated with RSV infection, post-viral adherence and IAV infection of Mϕs, resulting in NOX-2 oxidative burst [115,116]. With regard to SARS-CoV-2 infection in COVID-19 patients, severe disease is associated with more robust ROS production compared to mild disease [117]. Finally, the complement system is also an important component of innate defence and is involved in the immunopathology of and disease severity of SARS-CoV-2 infection. Carvelli et al. [118] found that COVID-19 severity was proportional to the inflammatory mediator C5a. C5a acts as a chemokine and can both recruit and activate neutrophils and monocytes expressing the C5a receptor, C5aR1, thus initiating and perpetuating a prolonged inflammatory response. Whilst the membrane attack complex (MAC: C5b-9) can be used to lyse infected cells, there seems to be no significant correlation between viral load and MAC complement activation, suggesting a role for the complement in inflammation alone [119]. Thus, the control or dysregulation of the inflammatory response is vital in determining appropriate host immunity to SARS-CoV-2 infection or immunopathology.

### 5.2. Adaptive Immunity

Host adaptive immune responses to viral infection also involve several phases, which include: (1) Antigen processing/presentation—MHC presentation of viral antigens; (2) Humoral immunity—Nabs (neutralisation of infection), immune complex-mediated mechanisms (possibly type III hypersensitivity); (3) Cell mediated immunity (CMI)—both activation of CD8^+^ Tc and CD4^+^ Th_1_ DTH responses; (4) Cytokines; priming and regulation of Th_1_, Tfh, Th_17_, Treg, and the effector responses of these cells (refer to Figure 1).

In general, as a consequence of their intracellular habitat, the host mounts a cell-mediated immune response characterized by either activation of Tc or Th1-dependent activation of pro-inflammatory Mϕs as part of the DTH. Indeed, IAV infection of mouse lung epithelial cells and human A549 cells has been observed to upregulate MHC class I molecules vital for antigen presentation, restricting Tc activation [120,121]. When focussing on SARS-CoV-2 infection, the host immune system mounts several adaptive responses, predominated by cell-mediated immunity. SARS-CoV-2 infection induces the proliferation and activation of highly cytotoxic CCR7^−^ CD27^+^ CD28^+^ CD127^-^ CD8^+^ Tc [122], which kill infected host cells via cell–cell killing mechanisms involving FasL and TRAIL and secretory mechanisms utilising perforin and granzyme. These killing mechanisms are suppressed as COVID-19 progresses from acute, moderate infection to one that is more severe and chronic [89], in which Tc exhaustion/tolerisation is characterized by elevated programmed cell death protein-1 (PD-1), CD244 and decreased perforin, and granzyme. Thus, the relative balance between immune activation and immune evasion/suppression responses plays a significant role in determining degree and progression of infection. In addition to Tc, cell-mediated immunity may also involve Th_1_-mediated activation of pro-inflammatory M1-subset Mϕs. These are dependent on cytokines such as IFNγ (produced by Th_1_ cells and NK cells) which induce a less-targeted inflammatory response, killing infected cells and resulting in collateral damage to surrounding host cells and tissue. Such tissue damage can serve as a positive feedback loop, creating a cycle of Mϕ activation and secretion of pro-inflammatory cytokines, which with appropriate regulation mediates antiviral immunity whereas in poorly regulated conditions, associates with chronicity and severe disease linked to characteristic cytokine profiles of CRS [123,124,125].

As a consequence of viral infection, persistence, and spread, SARS-CoV-2 not only resides inside host cells such as AMs/epithelial cells but also extracellularly. The immune system is capable of responding to extracellular SARS-CoV-2 by mounting a humoral response, resulting in B cell activation and the secretion of antigen-specific antibodies. Activated B cells can secrete several isotypes, which include IgM, IgA, and IgG, that exhibit differential immune responses to the virus [126], with mild-symptom COVID-19 exhibiting lower IgA and IgG titres compared to the S1 protein [127]. IgA, detected throughout the course of infection, and IgM are both mucosal antibodies that are present in mucosal secretions, which trap antigens/viral pathogens, effectively neutralising the infective capability of the virus. In addition, both IgM and IgG antibodies induce innate mechanisms resulting in viral neutralisation via the spike protein and complement activation [128], inflammation, and hypersensitivity responses, resulting in antibody-dependent cell cytotoxicity (ADCC) against spike protein-expressing target cells mediated by CD16^+^ NK cells [129,130]. These antibody responses collaborate to drive efficient virus neutralisation and clearance mechanisms. It must be noted, however, that antibody-dependent enhancement (ADE) has been observed to contribute to severity of SARS-CoV-2 infection, which may also affect vaccine efficacy. This has been suggested to be driven via binding of anti-S protein IgG to FcγRII^+^ cells, such as Mϕs, effectively enhancing both viral entry and the secretion of pro-inflammatory cytokines, which contribute to the CRS (reviewed in Garcia, 2020 [131]). Again, dysregulation of such responses may create a predisposition to over-exuberant responses, which result in damage to host tissue.

Immune hypersensitivity is associated with several viral infections, hence tissue-destructive pathological mechanisms are predominantly driven via host immune mechanisms rather than direct responses elicited by the virus. Dysregulation of such mechanisms can contribute to viral pathology, persistence, and evasion strategies employed by SARS-CoV-2. Lymphopenia has been associated with COVID-19, and several studies have reported lower numbers of T cells in infected patients [89,119]. It has been suggested that this might be influenced by the cytokine profile that accompanies SARS-CoV-2 infection, since TNF-α has been linked to T cell apoptosis [132]. Whilst innate responses seem to be more prominent in COVID-19 immunopathological mechanisms, it cannot be overlooked that these innate mechanisms can be influenced by products of the adaptive immune system. IL-17 is one such example, being produced by Th_17_ cells with the capability of inducing a positive feedback loop, resulting in neutrophil (Nϕ) expansion and activation [133]. Thus, not only can some T cell subsets be reduced in both numbers and function, but some Th subsets may be primed, resulting in an immune bias towards perpetuating systemic inflammation driven by a dysregulated cytokine storm or CRS.

### 5.3. Viral Immune Evasion Mechanisms

Upon recognition and induction of an antiviral immune response, many viral pathogens have established mechanisms by which these defensive responses are evaded. SARS-CoV-2 and many other related respiratory pathogens are no exception: they have acquired a multitude of mechanisms by which they evade host antiviral responses through immune suppression and deviation (see Table 1). SARS-CoV-2 and related respiratory pathogens utilise a multitude of immune escape mechanisms that are directed at both innate and adaptive responses of the host; these include suppressive or modulatory effects targeting viral recognition, its signalling, and antiviral responses including AMP production, type I IFN production and signalling, NK activation, CMI and antibody production, and downstream effector mechanisms focussed on both viral infection and activation of cytotoxicity.

**Table 1:** Coronavirus and respiratory viral immune evasion responses.

Viral Immune Evasion Response	Infection/Pathology	Reference
Recognition by PRRs	M inhibits RIG-1,MDA5, and MAVS 9b inhibits interaction between RIG-1 and MAVS Nsps-3,-4,-6 encode double-membrane vesicles, hiding dsRNA from RLRs N protein binds TRIM25, preventing RIG-1 act^n^	[134]
Type I IFN production and signalling	Nsp-1 degrades IFN mRNA Nsp-1blocks STAT-1 phosphorylation—delaying type I IFN production Nsp6 inhibits TBK1 phosphorylation of IRF7 7a destabilises TBK1, inhibiting IRF3 phosphorylation SARS-CoV-2 ORF6 inhibits downstream IFNα signalling Nsps-1,-3,-13,-14, ORFs-6,-8, M and N inhibit IFN type I-induced ISG gene expression N protein binds TRIM25, preventing RIG-1 act^n^ and reduction in IFNβ production IAV Nsp1 inhibits RIG-1-IFNβ production SARS-CoV-2 ORF3b—potent antagonist of IFN prod^n^ via suppression of IRF3 nuclear translocation	[134,135,136,137,138]
Anti-inflammatory cytokine production	Nsp3 ORF9b and M inhibit NFκB activation SARS-CoV-2 strongly induces AM IL-10 prod^n^	[88]
Suppression of MHC expression	MHC I expression inhibited by ORF3a and ORF7a SARS downregulates DC MHC II expression MERS downregulates Mϕ MHC II SARS-CoV-2 ORF8 downregulates T cell MHC I	[87,139]
Suppression of NK cell activation	NKG2A upregulation—inhibits NK-mediated cell cytotoxicity (also on CD8^+^ Tc) Increased IL-6 and IL-10 inhibit STAT-3-dependent NK activation Both IL-6 and IL-10 increase NKG2A expression	[140,141]
Inhibition of Cell-mediated immunity - Tc cytotoxicity - DTH Th1—Mϕ activation	SARS-CoV-2 ORF3B antagonism of IFN production. NKG2A upregulation—inhibits CD8^+^ Tc-mediated cell cytotoxicity (also on NKs) SARS downregulates DC MHC II and B7 expression Antigenic mutation of M protein	[100,142,143]
Inhibition of Humoral Immunity - Neutralising Ab - ADCC - IC-complement	Omicron variant—high mutational burden in S protein: increased Ab evasion. SARS downregulates DC MHC II and B7 expression	[144,145]
Inhibition of receptor binding	Flexible RBD in S trimers: RBD exposed in standing state. Lying state—RBD not exposed, hence reduced binding, infection and immunogenicity	[146]

***Abbreviations:*** PRRs, pattern recognition receptors; RLRs, RIG-1-like recptors; RIG-1, Mda-5; MAVS; NSPs, non-structural proteins; dsRNA, double-stranded RNA; TRIM25, Tripartite motif-containing protein 25; IFN, interferon; STAT-1,-3, signal transducer and activator of ttranscription-1,-3; TBK-1, Tank-binding kinase-1; IRF7, interferon regulatory factor-7; ORF, open reading frame; IAV, influenza A virus; NF-κB, nuclear factor-κB; AM, alveolar macrophages; DC, dendritic cells; NK, natural killer cells; NKG2A, natural killer receptor G2A; CMI, cell-mediated immunity; Tc; DTH, delayed type hypersensitivity; Mϕ, macrophage; ADCC, antibody-dependent cell cytotoxicity, IC, immune complex; TLR, toll-like receptor; Ab, antibody; RBD, receptor-binding domain.

Integral to immunity and immunopathogenesis to SARS-CoV-2 infection is the potential of this virus to affect the host microbiome and the consequent effects of dysbiosis in the host’s ability to respond to SARS-CoV-2. Early investigations comparing the microbiome associations with SARS-CoV-2 infection to healthy, un-infected subjects indicate dysbiosis occurring in both the intestinal and the lung/airway microbiomes, which, however, is not demonstrated in the oral microbiome. With regard to the gut/intestinal microbiome, bacterial diversity is reduced, reflected in a lower abundance of beneficial symbionts and higher proportion of opportunistic pathogens such as *Actinomyces*, *Rothia*, *Veillonella*, and *Streptococcus*. In addition, several microbes have been suggested to be bacterial biomarkers of COVID-19 dysbiosis, including *Intestinobacter*, *Fusicatenibacter*, *Actinomyces*, *Romboutsia* and *Erysipelatoclostridium*. These changes in the gut microbiome and resulting dysbiosis associated with COVID-19 correlate with clinical indicators elevated in the cytokine storm, namely CRP, IL-2, IL-4, IL-6, and TNF-α [147]. Conversely to the bacterial microbiome, the gut fungal mycobiome exhibits dysbiosis, displaying an increase in fungal diversity and enrichment of opportunistic fungal pathogens such as *Candida albicans*, *Auris candida*, and *Aspergillus flavus*. Studies investigating microbial populations in both bronchoalveolar lavage fluid (BALF) and nasopharyngeal swabs have demonstrated changes in the lung/airway microbiome. Patients with mild COVID-19 failed to show any significant differences in bacterial diversity and overall composition; however, patients with more severe COVID-19, particularly ICU-admitted SARS-CoV-2 infected patients, exhibited a disappearance of *Bifidobacterium* and *Clostridium* whereas *Salmonella*, *Scardovia*, and *Serratia* were detected [148].

To be able to adopt the use of probiotic bacteria in the prophylaxis and treatment of SARS-CoV-2 infection, it is imperative to consider the fine balance between appropriate host immune response, viral evasion strategies, and microbiome profiles resulting in immunopathological mechanisms. The optimal utilisation of probiotics will both consider strain-specific effects of oral administration and thus fine immune balance, but will also consider the integral relationship of these mucosal immune mechanisms linked via the gut–lung axis.

### 5.4. SARS-CoV-2 Infects and Affects Tissues/Organs Distal to the Gut–Lung Axis

Alongside the typical respiratory infection and the involvement of the gut discussed above, SARS-CoV-2 has been observed to cause complications at many distal sites which display high expression levels of ACE2, such as vascular complications, acute kidney injury, cutaneous manifestations, neuromuscular involvement, liver dysfunction, and reduced testicular spermatogenesis [94,149,150,151,152]. Multi-organ damage (MOD) has been described in many cases, with a high fatality rate in patients who develop MOD [153]. Owing to its essential role in angiotensin conversion for antihypertension and cardioprotection, crucial for normal organ function [154,155], ACE2 is found on many tissue types across the body. ACE2 expression levels were found to be highest in the small intestine, testes, kidneys, heart, thyroid, and adipose tissue, and were lowest in the blood, spleen, bone marrow, brain, blood vessels, and muscle. This systemic expression of ACE2 may explain the involvement of so many distal sites in complications of COVID-19 [156,157]. It does not fully explain, however, why some patients show such severe MOD and does not fully correlate with typical COVID-19 disease pathology, as respiratory tissue is not a strong expresser of ACE2 [157]. The cytokine storm may provide a further explanation for the development of distal site pathologies in COVID-19 disease progression. Associated with severe COVID-19 pathology, the cytokine storm (or CRS) is thought to be a key driver of distal site complications and MOD [153]. This overproduction of pro-inflammatory cytokines such as TNF-α, IL-6, IFNγ, and IL-1 leads to a destructive systemic inflammatory pathology causing significant cell death and organ damage [155,157]. This cytokine storm has been linked to patient outcome, with severely affected patients displaying heightened levels of cytokines, including IL-6, TNF-α and IFNγ, compared to patients with mild to moderate disease [125,158]. It must be considered, however, that long COVID causes prolonged symptoms ranging from shortness of breath to heart palpitations weeks, or even months, after viral clearance. This chronicity of localised and systemic inflammation can have a significant effect on tissue integrity and may even result in tissue remodelling. This systemic involvement in long COVID is associated with the inflammatory process and the ability of SARS-CoV-2 to disrupt the vascular endothelium of other organs such as the heart, kidneys, and brain.

## 6. Probiotics Reduce COVID-19 Symptoms of the Gut Mucosa via the Gut–Lung Axis

As discussed above, the gut microbiome and exogenous sources such as probiotics exert immunomodulatory effects that could protect the host against this cytokine storm and reduce MOD. The most severe cases of COVID-19 are classified as critical illness associated with septic shock and MOD (National Institute of Health, 2021). In COVID-19 patients, there was a strong association between hospitalised patients, COVID-19 disease severity, and gut dysbiosis, compared with healthy subjects and pneumonia disease-control patients [159]. As dysbiosis has a clear effect on the systemic immune response and impacts on disease progression, it stands to reason that the restoration and maintenance of the healthy gut microbiome by probiotics could potentially reduce the destructive inflammatory pathology associated with severe COVID-19 and thus prevent MOD. A meta-analysis conducted by Patra et al. (2021) [160] found that COVID-19 patients taking probiotics had significantly less severe symptoms. Although little research has so far been conducted on the direct impact of probiotics on MOD and systemic inflammation, research into similar pathologies implies the potential benefits of probiotics in this sense. In sepsis patients, where the pathogenesis also exhibits a cytokine storm with an overabundant/dysregulated immune response, dysbiosis of the gut microbiome was associated with this condition and aggravated sepsis-induced liver injury [161]. Rheumatoid arthritis (RA) is an autoimmune systemic inflammatory disease, primarily causing articular joint inflammation [162]. RA patients consuming probiotics containing *L. acidophilus*, *B. bifidum*, and *L. casei* were found to have a significantly improved disease score (i.e., reduced inflammation, tenderness, and swelling of joints), lower serum C-reactive protein (CRP), and inflammatory cytokines (TNF-α and IL-12) than RA patients taking a placebo [163,164]. From these studies, probiotics exert a strain-specific and selective anti-inflammatory effect on systemic inflammation and thus could be used to help mediate the pro-inflammatory cytokine storm in COVID-19 and systemic effects on tissues distal to the gut–lung axis.

Whilst the usual symptoms linked to SARS-CoV-2 infection are fever, dry cough, and fatigue, quite a lot of patients also experience gastrointestinal (GI) symptoms. These include nausea, vomiting, and diarrhoea. A study run by Jin et al. (2020) [165] determined that 11.4% of COVID-19 patients admitted to hospital experienced GI symptoms, with diarrhoea being the most common. An increased incidence of ARDs was also reported in severe groups presenting with GI symptoms. Probiotic administration modulates the microbiome balance in the intestinal tract, enhancing mucosal barrier integrity and mucin production and have also been linked to decreased production of pro-inflammatory cytokines such as IL-1, IL-6, IFNγ, and TNF-α [44]. The gut microbiome effectively aids the immune response against various pathogens, including those of the respiratory tract.

In a study by Brown et al. (2017) [166], germ-free mice exhibited an increased vulnerability to respiratory pathogens, further indicating the existence of a gut–lung axis. Antibiotics were administered to mice before inoculation with *S. pneumoniae* and *K. pneumoniae*; these mice had defects in bacterial clearance, linked to a reduction of innate molecules expressed in the lungs, such as GM-CSF, CXCL2, and CXCL1, all of which contribute to Nϕ and Mϕ development and Nϕ recruitment. Whilst this study focused on bacterial lung infections, Mϕs play a role in the immune response against SARS-CoV-2. Mϕs found in the alveolar cavities of COVID-19 patients contribute to the characteristic cytokine storm (CRS), as well as clearing apoptotic cells [89,110]. In fact, a reduction of GM-CSF could lead to decreased Mϕ activity, or even differential polarisation of the Mϕ subset, in the lungs [110]. An investigation monitoring the prophylactic effect of probiotics on preventing influenza infections found that orally administered probiotics, taken for 12 weeks, significantly elevated IFNγ and secretory IgA (sIgA) levels as well as resulting in a lower incidence of disease [167]. IFNγ can induce B cells to produce antibodies, which stimulate the complement system as well as neutralising binding activity/infectivity of pathogenic microorganisms. This suggests that both cell-mediated- and humoral-adaptive immunity had been boosted, beneficial when priming a SARS-CoV-2 immune response.

As with the lungs, the ACE2 receptor is present in the gut, expressed by epithelial cells of the small intestine. This allows SARS-CoV-2 to interact directly with and infect the gut mucosa. It has also been theorised that the virus can affect the gut mucosa indirectly via the gut–lung axis, suggesting that both organs share a common mucosal immune system and that disturbances in one can affect the other [168]. Upon infection, CCR9 is expressed on lung mucosal T helper (Th) memory cells, which can migrate to gut epithelial cells expressing CCL25. Of particular relevance, CCL25 can also recruit Mϕs, DCs, IELs, and IgA-secreting plasma cells, all of which are integral to mucosal defence (refer to Figure 1 below).

A disruption to tryptophan absorption could be responsible for increased cases of diarrhoea, since the ACE2 receptor plays a role in amino acid homeostasis [168]. Tryptophan is absorbed by the gut via the B0AT1/ACE2 transport pathway and metabolised by the intestinal microbiome. Insufficient levels of this amino acid have also been linked to the development of colitis. Whilst the long-term implications of SARS-CoV-2 infection are currently unknown, it is not completely unreasonable to suggest colitis as a potential future consequence. Dysbiosis of the gut microbiome has been linked to Th_17_ polarisation in influenza infections [169]. These lymphocytes produce IL-17 which recruits Nϕs, contributing to inflammatory damage, observed in both lung and gut pathology. Antibiotics can also cause diarrhoea by disrupting the microbiome and have been administered to COVID-19 patients to treat secondary bacterial infections [170], hence further contributing to gut mucosal symptoms as a consequence of COVID-19-linked opportunistic pathogen infection. Thus, probiotic use to redress microbiome dysbiosis may restore tryptophan availability and regulate Th_17_/Nϕ-mediated pathology associated with both colitis and COVID-19.

Whilst vaccine development has been very successful in prophylaxis to viral infection, currently there is no specific direct antiviral treatment against SARS-CoV-2 infection. Since the microbiome produces immune modulators and vitamins, using probiotics to increase and improve the microbiome might be an effective prophylactic route, considering these products are natural immune enhancers/regulators with minimal to no side effects [44]. It must be noted that one probiotic strain cannot benefit all infections or all SARS-CoV-2-induced pathological mechanisms universally; studies are required to determine the best probiotic cocktail for both prophylaxis and real-time treatment of COVID-19. In addition, different combinations of probiotic bacteria could also affect people differently, depending on the current makeup of an individual’s microbiome, which is suggestive of a personalised medicine approach via manipulation of this gut–lung axis.

### 6.1. The Potential Role of Probiotics in the Prophylaxis of SARS-CoV-2 Infection and Pathology

Modulation of the initial innate immune response to early infection via probiotics could therefore be highly beneficial in preventing the development of both innate and adaptive COVID-19-associated immune response complications, such as ARDS or secondary infections such as pneumonia. The prophylactic ingestion of probiotic formulations, due to their modulatory effects on innate immunity, may serve to both encourage protective antiviral responses and dampen harmful over-exuberant inflammatory responses mounted by the host. This, however, is a difficult balance to be struck, considering the nature of innate immune signalling and its role in adjuvanticity required to prime and enhance antigen-specific protective immunity encouraged through vaccination. Indeed, probiotics have been used in China as a form of prophylaxis to prevent secondary bacterial infections, which can increase mortality in COVID-19 patients [171].

ACE-2 receptors are expressed in the inferior pylorus, antrum, and corpus sections of the stomach as well as other GIT sites, such as the small intestine and colon [172,173]. Stimulation of these receptors initiates several pro-inflammatory immune cascades, resulting in inflammasome assembly and hence IL-1β and IL-18 secretion and induction of type-1 IFN as well as expression of NFκB-dependent cytokines. Downstream of pro-inflammatory molecule (prostaglandins, VEGF, NFκB, TNF-α, IL-1β, IL-6 and IFNγ) production, chemokine-mediated recruitment of inflammatory immune-modulatory cells such as monocytes, Nϕs, and the downstream differentiation and stimulation of both M1 and M2 Mϕ subsets occurs [153]. As the M1 Mϕ is required for viral infection resolution and M2 for the prevention of and repair to tissue damage, it is important to maintain a delicate balance between the two to optimise viral clearance. Dysbiosis can result in an M1 Mϕ predominance via direct polarisation or plasticity changes from M2 Mϕs to M1s, resulting in incomplete viral clearance. The impact of the protective gut microbiome on this Mϕ balance, through the bacterial wall-derived lipopolysaccharides (LPS) in particular, significantly affects the structural integrity of the gut wall [93]. Therefore, being aimed at the gut flora found in these ACE-2 receptor-rich regions, suitable probiotics may improve immune protective strength and maintain gut barrier integrity against SARS-CoV-2, preventing viral infection/replication and symptom development. Alteration of the GIT microbiome is also known to affect other distal mucosal or ACE-2-exhibiting sites such as the lungs, heart, and kidneys, reducing the systemic inflammatory response initiated [92]. Two probiotics found in fermented milk products, *Lactococcus lacits* and *L. helveticus*, could implement this immunomodulatory capability to distal sites, effectively reinforcing the gut–lung axis of immune protection [92]. It is important to consider that any reduction in ACE-2 receptor expression as a form of prophylactic treatment would have to be recovered after viral exposure/infection, as permanent reduction may lead to a deterioration in cognitive ability [174]. One such recovery probiotic strain that may rescue ACE-2 receptor expression is *L. paracasei*; thus, an increase in ACE-2 receptors, through probiotic intervention, is being explored as a treatment option [175], but would have to be carefully managed to prevent overexpression, hence increasing SARS-CoV-2 infectivity.

Another beneficial probiotic mechanism, which may induce a more protective response, is the inhibition of pro-inflammatory cytokines, including TNF-α. Previously, this cytokine suppression approach has been used for the control and treatment of inflammatory diseases, such as RA and inflammatory bowel diseases (IBD), the sites of involvement of which have been suggested to be linked by a gut–joint axis similar to the gut–lung axis important for establishment of probiotic treatment of SARS-CoV-2 infection. When considering a preventative treatment for COVID-19, however, the probiotic genus *Lactobacillus* has been observed to have a significant effect upon this cytokine. When investigating the impact of TNF-α suppression on viruses, particularly influenza, the research is often conflicting, both advocating and contradicting the proposed intervention. Some suggest that TNF-α is essential for a strong innate antiviral defence, as it inhibits influenza virus replication, whereas anti-TNF treatment of HIV-1-infected patients not only reduces symptoms in these immunocompromised patients but also reduces the viral reservoir [176]. Thus future probiotic-mediated prophylaxis, via management of TNF-α, will have to carefully consider TNF-α modulation and probiotic strain utilized. It is well-established that mass stimulation of pro-inflammatory cytokine production, also known as the ‘cytokine storm’ elicited by SARS-CoV-2 infection, is the predominant cause of patients developing acute respiratory distress syndrome (ARDS), leading to the requirement for intensive care treatment. Collateral tissue damage, caused when some patients develop ARDS, occurs not via the virus itself, but as a result of a dysregulated hyper-inflammatory host immune response to eradicate the virus. Recruitment of Mϕs, Nϕs, and lymphocytes and activation of NLR-family receptors alter the respiratory mucosal vascular endothelium and that of the gut in an attempt to clear the virus [177]. Stimulation of the Nod-like receptor pyrin domain-containing 3 (NLRP3) inflammasome pathway facilitates pro-cytokine processing resulting in secretion of IL-1β and the downstream induction of IL-6. As with TNF-α, these destructive pro-inflammatory cytokines aid to clear the virus; however, through their destructive mechanisms, also damage the host. One such mechanism to reduce the amount of unnecessary pro-inflammatory cytokine production could be through probiotic NLRP3 inflammasome inhibition [178]; indeed, *Enterococcus faecalis* downregulates the production and assembly of this NLRP3 inflammasome, with a consequent reduction in the secretion and activity of IL-1β and IL-18 [179]. Such prophylactic treatment may reduce the severity of disease and systemic inflammatory complications but must be cautiously adopted to avoid suppression of beneficial protective anti-microbial responses. Therapeutic suppression of IL-6 may reduce the inflammatory response by inhibiting VEGF, adhesion molecule expression and influence vascular permeability. IL-6 expression is positively regulated by TNF-α, which acts as an apex cytokine; thus, targeting TNF-α may have a knock-on effect on several other downstream pro-inflammatory cytokines of the cytokine storm/ARDS, such as IL-6 [180]. A murine study investigating the impact of probtioics on IBD (characterised by dysbiosis of the microbiota, associated with the adoption of a pro-inflamamtory state), found that upon administration of a probiotic cocktail of *Bifidobacteria*, *Lactobacilli*, and *Streptococcus thermophilus* DSM24731, pro-inflammatory cytokines TNF-α/IL-6/IL-1β decreased, and anti-inflammatory cytokines, such as IL-10, increased. In contrast, *L.rhamnosus*, can attenuate IL-6 production, particularly in those who already have a damaged GIT. Therefore, probiotic recommendation should potentially be administered on a case-by-case basis [181].

SARS-CoV-2-infected individuals can be susceptible to bacterial invasion and hence prone to developing secondary opportunistic infections, such as pneumonia. Lipopolysaccharide (LPS) is usually found in Gram-negative bacterial walls and recognised by TLR4 [182]; despite SARS-CoV-2 not containing LPS, and thus not being directly recognised by TLR4, stimulation of this TLR is associated with the development of ARDS in COVID-19 patients, demonstrating the impact of bacterial stimulation on patient symptoms. This not only was confirmed by a study in which mice given ventilation support increased their risk of secondary bacterial infection, but also suggests that TLR4 could indirectly be a potential therapeutic target, via treatment with TLR4 antagonists [183]. Probiotic intervention can significantly alter TLR expression both extracellularly and intracellularly; *L.acidophilus*, for example, upregulates TLR-2, aiding innate viral defence mechanisms [184]. A strategic immuno-evasive strategy employed by COVID-19, which successfully reduces host immunity, is through blocking viral-specific TLRs such as TLR7 and TLR3 (see Table 1). A potential therapeutic method adopted for both prophylaxis and post-infection treatment could therefore be upregulation of these receptors [183]. Indeed, *L. rhamnosus* increases expression of TLR3, which targets double-stranded viral DNA and only appears in COVID-19 patients once the virus has replicated. Another issue with probiotic upregulation of receptors is that an increase in TLR7 as well as TLR8 can become prolonged, leading to uncontrolled immune activation and inflammation progressing to severe immunopathology. Again, careful consideration of probiotic strains and administration is required for adoption as prophylaxis against SARS-CoV-2 through the manipulation of TLR expression.

**Figure 2:**
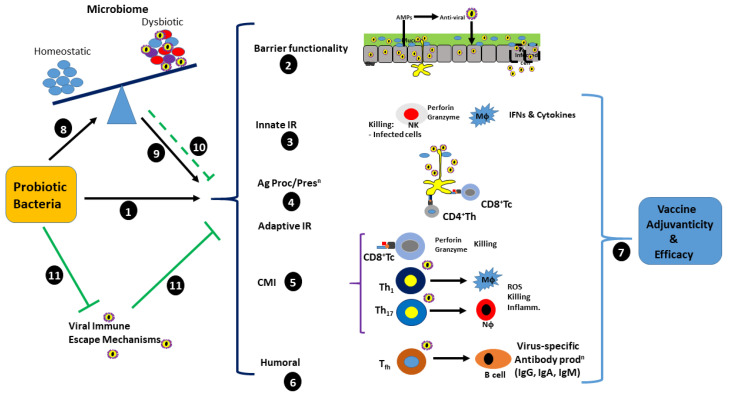
**The potential of probiotic bacteria to initiate and augment IR either directly or indirectly via modulation of the gut microbiome**. Probiotic bacteria positively stimulate immunity (1) via several mechanisms, which include (2) enhancement of barrier functionality (AMP secretion, mucin expression, and cytokine production), (3) activation of NK cell killing, antiviral IFN responses, and Mϕ inflammation, (4) Ag processing/presentation via MHC I, II, and B7 expression and Ag-specific adaptive immunity responses by (5) CMI—Tc, DTH and Th_17_ responses as well as (6) Tfh-mediated B cell humoral responses, producing virus-specific Abs, capable of initiating complement activation, ADCC and neutralisation of infectivity. Probiotic initiation of 3–6 facilitate (7) adjuvanticity and efficacy of anti-S protein SARS-CoV-2 vaccines. Probiotic bacteria can also affect immune responsiveness indirectly by (8) influencing the microbiome, where a homeostatic microbiome positively (arrow—black, pointed) influences antiviral immunity (9), and dysbiosis potentially suppresses (blunted green line) antiviral immunity (10). Finally, probiotic bacteria may suppress viral immune escape mechanisms by which SARS-CoV-2 evades host immune protection (11).

In summary, knowledge of host immunity to respiratory viral infections such as SARS, MERS, IAV, and RSV, along with an understanding of viral escape mechanisms and how both are potentially modulated by probiotics, affords us an understanding of how probiotic bacteria may be harnessed to enhance prophylactic and post-infection treatment (see Figure 2). With respect to prophylaxis, probiotics may be used to decrease SARS-CoV-2 infectivity via suppression of ACE-2 receptors and the induction of TLR3, TLR7/8 expression, and Nϕ AMPs capable of inhibiting viral binding to ACE-2 receptors. This prophylaxis may also be reinforced by a selective stimulation of the inflammasome, resulting in IL-18 production and its downstream involvement in activation of NK cells and CMI, mediated by CD8^+^ Tc and Th_1_-activated Mϕs. The additional capability of probiotics to induce humoral immunity resulting in antibody secretion only further reinforces a prophylactic approach by inducing an antiviral response which includes innate, CMI, and humoral mechanisms required and initiated by some of the most efficient vaccines currently being used, which target the spike protein. Overall, prophylactic control of SARS-CoV-2 infection throws up many challenges with respect to mechanistic approaches. This may incorporate methods, seeking to reduce immune responsiveness, hence tolerating viral infection and simultaneously inhibiting the damaging effect of over-exuberant host responses or via the selective upregulation of innate antiviral immune responses with minimal collateral damage to host tissues.

### 6.2. The Potential Role of Probiotics in the Treatment of SARS-CoV-2 Associated Secondary Infection

In addition to modulating viral prophylaxis, probiotics may represent a useful approach to the direct treatment of established SARS-CoV-2 infection and complications, such as those posed by secondary infection. Again, this will have to consider a delicate balance between initiating appropriate host immunity without over-activation resulting in immune-mediated pathology. When considering the long-term related conditions associated with SARS-CoV-2 infection and those that require a more delayed/longer-lasting, specific humoral and cell-mediated immune response to fight infection, the involvement of opportunistic bacteria increases. Lower respiratory tract infection, which can result in pneumonia in COVID-19 infected patients, cannot only be caused by viral infection but also through secondary bacterial infection. This is primarily due to the immune response being weakened or preoccupied due to the already existing viral infection and its consequent immune-evasion mechanisms, allowing opportunistic pathogens to invade and proliferate. Damage to the mucosal layer, as seen in influenza virus infections as well as in SARS-CoV-2 infection, allows bacterial adherence of *S. pneumoniae*, *Pseudomonas aeruginosa*, and *Haemophilus influenzae* and thereby changes the bacterial flora found in these mucosal surfaces [2]. *S.pneumoniae* is a commensal usually found in the nasopharynx and only becomes opportunistic when it moves to other mucosal surfaces, such as the lungs [185]. Intubation of SARS-CoV-2-infected patients can result in bacterial translocation, where the bacteria are forced down into the trachea. *S.pneumoniae* can be catastrophic due to its ability to also invade the bloodstream and cause sepsis [186]. Prevention of this bacterial infection is therefore crucial, particularly in the immunocompromised or those at an increased risk of contracting COVID-19.

The strong epithelial cell adherence of *L. rhamnosus GG* (LRGG) prevents *S.pneumoniae* proliferation, colonisation, and invasion of the internal lung surface without affecting the cytokine balance within the immune system, allowing the body to continue to fight the viral infection whilst these two bacteria have their relatively private battle [187]. This may or may not, however, prove to be beneficial when considering the adverse effect SARS-CoV-2 exerts on these immune-signalling molecules via an induced cytokine storm or CRS. An investigation utilising the probiotics LRGG, *Bacillus subtilis*, and *Enterococcus faecalis* revealed that SARS-CoV-2-infected patients were significantly less likely to develop ventilator-associated pneumonia and respiratory tract infection [188,189]. LRGG’s ability to inhabit lung epithelial cells and maintain gut structural integrity through the gut–lung axis, and thereby reduce gut-associated inflammatory issues, is yet to be proven to have an effect upon the severity of SARS-CoV-2 infection. Daily administration of *Bifidobacterium breve* has also been suggested to reduce the risk of ventilator-associated infection, whereas LRGG should be taken once throughout the whole infection. These recommendations, however, are based upon other viral infections and not SARS-CoV-2 [190]. SARS-CoV-2 infection has been shown to persist longer within the GIT, particular in faecal matter, than in the lungs, demonstrating the importance of maintaining gut wall structural integrity [191]. The consistency of the stool is also another indicator of the severity of SARS-CoV-2 infection, with a positive correlation found between viral severity and diarrhoea looseness and release rate. Probiotics again can have an impact upon symptom severity here; their ability to prevent bacterial infection related diarrhoea is more profound in children than in adults, with strains such as LRGG and *Saccharomyces boulardii* decreasing the duration of diarrhoea by an average of one day [192].

Probiotics can thus potentially play an important role in protection against SARS-CoV-2 infection. They generally act in a strain-dependent manner, capable of modulating antiviral responses at several levels, including maintaining or redressing homeostatic microbiome balance, inducing antiviral responses of mucosal barriers (AMP, Ab-neutralisation), innate immunity (NK killing, Nϕ and Mϕ inflammation), adaptive immunity (both CMI-Tc and DTH and humoral Ab-mediated responses (ADCC, neutralisation and complement activation) as well as suppression of viral immune escape mechanisms (refer to Figure 2). The careful selection and formulation of probiotic strains and mixtures of strains may thus act prophylactically, reducing SARS-CoV-2 infection and spread, as well as at a treatment level upon infection to help reduce pathogenic responses triggered by the virus, secondary bacterial infection, and the host in response to the virus and opportunistic pathogen. This wide array of effects on antiviral immunity may thus also enhance vaccine efficacy via immune-boosting adjuvant activity as well as antigen-specific tailoring immunity via appropriate immunomodulatory effects.

The potential for probiotics to positively impact antiviral immunity against SARS-CoV-2 infection and viral-induced pathology is being pursued vigorously in several clinical trials, which both target prophylaxis and treatment of established infection and disease. Thus far, there are 33 reported COVID-19/probiotic clinical trials reported by NCT; of these trials, 7 are recruiting, 2 are not recruiting, 1 has been terminated, 4 are active-not recruiting, and 1 has been suspended. There are 15 trials, however, that have been completed, with 1 trial reporting preliminary results [193,194,195,196,197,198,199,200,201,202,203,204,205,206,207] (see Table 2). Indeed, NCT04507867 reported a level of tolerability with a potential to regulate inflammatory markers such as CRP [207]. This indicates that probiotics may play a therapeutic role in suppression of harmful host-mediated inflammatory symptoms associated with COVID-19 pathology. Future investigations are likely to result in adoption of specific strains and mixtures of probiotic strains in the management of SARS-CoV-2 infection and COVID-19 pathology: being involved in both prophylaxis (protection preventing infection) and treatment of established infection. 

## 7. Summary and Conclusions

SARS-CoV-2 infection can drive both an acute and chronic disease, characterised by tissue damage driven by an over-activated inflammatory response that manifests in both localised respiratory tissue and systemic tissues, distal to the lungs. With the establishment of a gut–lung axis of viral infection, the balance of the intestinal microbiome may play an important role in immune fate decisions behind viral clearance or virus-induced immunopathology. The association of microbial dysbiosis in the gut with SARS-CoV-2-driven lung pathology effectively opens up the possibility of managing this infection by redressing the balance of the microbiome by adopting an approach mediated by probiotics. As such, this potential of probiotics to manage COVID-19 and SARS-CoV-2 infection is being investigated in current national clinical trials (NCTs) employing single strains of *Lactococcus lactis* W136 and *Lactobacillus* (plantarum and coryniformis K8) or mixtures of *Lactobacillus* and *Pediococcus acidilactici* and even washed microbiome transplantation (NCT04366180, NCT04517422, NCT044585, NCT04458519, and NCT04251767, reviewed in [208,209]). To summarise, the use of probiotics may significantly improve the outcome of COVID-19 patients in a multitude of actions. They have potential use as a mediator of the immune response during COVID-19 through the gut–lung axis and systemic immune modulation as well as in a restorative capacity after infection to repair immune-inflammatory damaged tissue and redress the dysbiotic microbiome. Furthermore, they may act as a prophylactic preventative for SARS-CoV-2 infection and more severe disease by promoting antiviral immunity and viral clearance through immune activation. Probiotics exert a myriad of antiviral responses, which include neutralisation of virus infectivity and both innate and antigen-specific adaptive responses harnessing both cell-mediated and humoral antiviral immune mechanisms. These probiotic-driven antiviral responses, however, would appear to be both strain-specific and dependent on the strength and adaptability of the immune response relative to the highly developed evasion strategies employed by SARS-CoV-2. Utilising this knowledge, it would appear that probiotics, mixtures of probiotic strains and synbiotics (probiotics + prebiotics) may be harnessed to utilise these microorganisms as ingestible adjuvants and immune modulators capable of strengthening natural prophylactic responses, vaccine-induced memory responses and the treatment of acute or chronic viral infection, secondary opportunistic pathogen infection and its resulting devastating inflammatory immunopathology.

## Figures and Tables

**Table 2:** **Completed clinical trials involving probiotic treatment and management of COVID-19.**

Study Ref. Clinical Trial Identifier	Study Title Study Focus	Probiotic Intervention	Procedure Synopsis	Country & Start/Completion of Study [Reference]
NCT05080244 No results	Probiotics: reduce occurrence of long COVID-19	ProB—2 strains (ProB strains not disclosed)	618 patients, RCT. 2 capsules/d for 10d, then 1/d to day 25.	Quebec, Canada Oct 2021/Dec 2022. [193]
NCT04621071 No results	Probiotics: reduce duration & symptoms of COVID-19	ProB—2 strains (ProB strains not disclosed)	17 patients, RCT. 2 capsules/d for 10d, 1 capsule/d to day 25.	Quebec, Canada Jan 2021/Oct 2021. [194]
NCT05474144 No results	Efficacy of ProB in patients with severe COVID-19 infection	SmartProbio C 19 strains + inulin + maltodextrin	83 patients, RCT. Triple masking. Twice/d, 2 wks.	Brno, Czech Republic. Nov 2021/April 2022. [195]
NCT04390477 No results	Evaluate effect of ProB in COVID-19	ProB strains (not disclosed) & maltodextrin	41 participants No masking. 1 capsule per day for 30 d.	Alicante, Spain May 2020/March 2021. [196]
NCT04458519 No results	i/n ProB reduct^n^ of symptom severity in COVID-19	Probiorinse *L.lactis* W136	23 participants, single blinded, for 14d	Montreal, Canada July 2020/May 2021 [197]
NCT04937556 No results	ProB supplement^n^ in IR of COVID-19 participants	*L. salivarius*, Vit D + Zinc	41 participants randomised triple masking, 28 d	Madrid, Spain Oct 2021/March 2022. [198]
NCT04734886 No results	ProB supplement^n^ on SARS-CoV-2 Ab IR after COVID19	*L.reuteri* DSM17938 + Vit D	161 participants Quadruple masking, daily for 6 months	Orebro, Sweden Nov 2020/Sept 2021. [199]
NCT05043376 No results	ProB S. salivarius K12 for hospitalised (non-ICU) patients with COVID-19	BLIS K12 *Streptococcus salivarius* K12.	50 participants randomised, open label. Daily, to day 14.	Lahore, Pakistan Sept 2021/Nov 2021. [200]
NCT05175833 No results	Oral ProB and secondary bacterial pneumonia in severe COVID-19	Oral gel ProB *Streptococcus salivarius* K12 & *L.brevis* CD2	70 participants Randomised, quadruple masking, 7 d course	Passo Fundo, Brazil Sept 2020/Jan 2021. [201]
NCT04847349 No results	Live microbials to boost SARS-CoV-2 immunity	Dietary supplement OL-1 (ProB consortium, strains not disclosed)	54 participants Randomised Quadruple masking, daily 21 d	New Jersey USA April 2021/Jan 2022. [202]
NCT04462627 No results	Reduction of COVID-19 transmission to healthcare professionals	Dietary supplement probiotic (Probactiol Plus—Metagenics) *B.lactis* Bi-07; *L.acidophilus* NCFM	566 participants, open-label, no masking.	Brussels, Belgium April 2020/April 2022. [203]
NCT04798677 No results	Efficacy & tolerability of ABBC1 in volunteers receiving influenza or COVID-19 vaccine.	ABBC1: beta-1,3/1,6-glucan + inactivated *Saccharomyces cerevisiae* (with Se, Zn)	72 participants RCT, triple masking. 30 d supplement^n^	Barcelona, Spain Oct 2020/Sept 2021. [204]
NCT04517422 No results	Efficacy of *L.plantarum* & *P.acidilactici* in adults with SARS-CoV-2 & COVID-19	*L.plantarum* CECT30292, CECT7484, CECT7485y & *P.acidilactici* CECT7483 with maltodextrin.	300 participants. RCT, quadruple masking. 1 dose per day over 30 d dietary supplement^n^	Mexico City, Mexico Aug 2020/Feb 2021 [205]
NCT04399252 No results	Effect of *Lactobacillus* on the microbiome of household contacts exposed to COVID-19	*L.rhamnosus* GG	182 participants. RCT, triple masking. 2 capsules per day over 28 d.	North Carolina, USA June 2020/July 2021. [206]
NCT04507867 Results available.	Effcect of a NSS to reduce complications in patients with COVID-19 and comorbidities in stage III.	Nutritional Support System: i/m Vit B1,B6,B12. *Saccharomyces boulardii* CNCM I-745 “Floratil”	80 participants. RCT, triple masking. 1 capsule, twice a day over 6 d.	Mexico State, Mexico. Sept 2020/April 2021. [207]

***Abbreviations:*** NCT, national clinical trial; ProB, probiotic; d, days; i/n, intranasal; i/m, intramuscular; RCT, randomised controlled trial; ICU, intensive care unit; NSS, nutritional support system; Vit, vitamin; Reduct^n^, reduction; Supplement^n^, supplementation; IR, immune response.

## Data Availability

The data presented in this study are both available on request from the corresponding author and contained in this manuscript.
